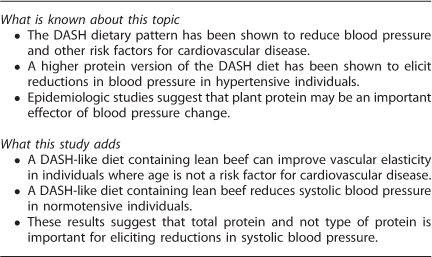# Effects of a DASH-like diet containing lean beef on vascular health

**DOI:** 10.1038/jhh.2014.34

**Published:** 2014-06-19

**Authors:** M A Roussell, A M Hill, T L Gaugler, S G West, J S Ulbrecht, J P Vanden Heuvel, P J Gillies, P M Kris-Etherton

**Affiliations:** 1 Department of Nutritional Sciences, Pennsylvania State University, University Park, PA USA; 2 Department of Statistics, Pennsylvania State University, University Park, PA USA; 3 Department of Biobehavioral Health, Pennsylvania State University, University Park, PA USA; 4 Department of Medicine, Pennsylvania State University, University Park, PA USA; 5 Department of Veterinary and Biomedical Sciences, Pennsylvania State University, University Park, PA USA; 6 New Jersey Institute for Food, Nutrition and Health, Rutgers, The State University of New Jersey, New Brunswick, NJ USA; 7 Present Address: Division of Health Sciences, University of South Australia, Adelaide, South Australia Australia; 8 Present Address: Department of Statistics, Carnegie Mellon University, Pittsburgh, PA USA

**Keywords:** Risk factors

## Abstract

A DASH (dietary approaches to stop hypertension) dietary pattern rich in fruits and vegetables and low-fat dairy products with increased dietary protein provided primarily from plant protein sources decreases blood pressure. No studies, however, have evaluated the effects of a DASH-like diet with increased dietary protein from lean beef on blood pressure and vascular health. The aim of this study was to study the effect of DASH-like diets that provided different amounts of protein from lean beef (DASH 28 g beef per day; beef in an optimal lean diet (BOLD) 113 g beef per day; beef in an optimal lean diet plus additional protein (BOLD+) 153 g beef per day) on blood pressure, endothelial function and vascular reactivity versus a healthy American diet (HAD). Using a randomized, crossover study design, 36 normotensive participants (systolic blood pressure (SBP), 116±3.6 mm Hg) were fed four isocaloric diets,: HAD (33% total fat, 12% saturated fatty acids (SFA), 17% protein (PRO), 20 g beef per day), DASH (27% total fat, 6% SFA, 18% PRO, 28 g beef per day), BOLD (28% total fat, 6% SFA, 19% PRO, 113 g beef per day) and BOLD+ (28% total fat, 6% SFA, 27% PRO, 153 g beef per day), for 5 weeks. SBP decreased (*P*<0.05) in subjects on the BOLD+ diet (111.4±1.9 mm Hg) versus HAD (115.7±1.9). There were no significant effects of the DASH and BOLD diets on SBP. Augmentation index (AI) was significantly reduced in participants on the BOLD diet (−4.1%). There were no significant effects of the dietary treatments on diastolic blood pressure or endothelial function (as measured by peripheral arterial tonometry). A moderate protein DASH-like diet including lean beef decreased SBP in normotensive individuals. The inclusion of lean beef in a heart healthy diet also reduced peripheral vascular constriction.

## Introduction

Atherosclerotic cardiovascular disease (CVD) is a multifactorial disease. Estimates indicate that more than 82 million American adults (1 in 3) have one or more types of CVD.^1, 2^ Many individuals in all ethnic populations have multiple risk factors for CVD, and the number of risk factors in individuals without diagnosed CVD is increasing.^1^ The major risk factors include smoking status, elevated body weight, total cholesterol, low-density lipoprotein cholesterol, blood pressure (BP) and fasting glucose.^1^ Approximately 60% of Caucasian adults and 80% of African American adults have at least one risk factor. DASH (dietary approaches to stop hypertension) is the ‘gold standard’ dietary pattern recommended by the American Heart Association,^3^ American Society of Hypertension^2^ and 2010 Dietary Guidelines for Americans^4^ for reducing many of these major CVD risk factors including abnormal lipids and lipoproteins, high BP, overweight/obesity and elevated blood glucose levels.^5, 6^ The DASH dietary pattern is reduced in saturated fatty acids (SFA), with emphasis on dietary carbohydrate from fruits, vegetables and whole grains; multiple minerals (potassium, magnesium and calcium) and fibre are also increased.

In addition to the major risk factors for CVD, there are other risk factors including those related to vascular health (endothelial function, vascular reactivity and so on). Our understanding of how diet affects vascular health is still evolving and more information is needed.

Individuals are often advised to avoid or restrict beef because it is a source of saturated fat in the diet. However, many Americans enjoy beef, commonly choosing cuts deemed lean by United States Department of Agriculture (USDA), and report better adherence to dietary advice that includes some lean beef.^7^ In addition, beef’s contribution to SFA in the American diet is often overstated in that it is not one of the top five contributors of SFA for Americans.^4^ In the BOLD Study, we showed that the inclusion of lean beef (4.0 or 5.4 oz per day) in a DASH-like diet decreased total cholesterol and low-density lipoprotein cholesterol similarly to the DASH diet.^8^ The DASH diet guidelines suggest reducing red meat as a strategy for controlling saturated fat;^9^ however, little is known about the effects on vascular health when lean beef is incorporated in a DASH diet.

In the present study conducted with normotensive individuals, we evaluated the effects of a traditional DASH diet as well as a DASH-like diet containing lean beef (beef in an optimal lean diet (BOLD); 113 g beef per day) and a moderate protein diet containing lean beef (BOLD+; 153 g beef per day) compared with a healthy American diet (HAD) as the control on vascular health, a secondary end point in the BOLD Study.

## Subjects and methods

### Subjects

The methods used for this study have been described in detail previously.^8^ Nonsmoking normo- or pre-hypertensive (BP <140/90 mm Hg) men and women (30–65 years) with moderately elevated low-density lipoprotein cholesterol (110–176 mg dl^−1^) were recruited.^8^ Additional inclusion criteria were body mass index (18.5–37 kg m^−2^) and fasting triglycerides <350 mg dl^−1^. Participants taking prescribed BP-lowering medication were eligible as long as their BP was below the exclusion criteria (one participant on BP medication was enrolled in the trial but excluded from the vascular health analyses). Exclusion criteria were: established CVD, stroke, diabetes, liver, kidney or autoimmune disease, the use of cholesterol/lipid-lowering medication or supplements (psyllium, fish oil, soy lecithin and phytoestrogens), being pregnant or lactating, experiencing weight loss of ⩾10% of body weight within the 6 months before enrolling in the study and vegetarianism. The Institutional Review Board at The Pennsylvania State University approved the experimental protocol, and all subjects provided written informed consent. This study is registered at ClinicalTrials.gov NCT00937898.

### Study design

The study employed a four-period, randomized, crossover, controlled-feeding design. Subjects were randomly assigned to a treatment (diet) order, and consumed each diet (HAD, DASH, BOLD and BOLD+) for 5 weeks. The diet periods were separated by a brief compliance break (average 1 week). On two consecutive days at the beginning of the study (baseline) and at the end of each diet period, participants completed a series of clinical and physical assessments (blood draw, height and weight) at the General Clinical Research Center of The Pennsylvania State University. The initial participants were enrolled in the study in September 2007; the final participants completed the study in March 2009.

### Diets

The composition of the experimental diets is presented in Table 1. The Harris–Benedict equation was used to estimate each participant’s energy needs, participants were monitored (daily weigh-ins) to verify they remained weight stable and calorie adjustments were made in 100 kcal increment to assure that weight remained stable for the duration of the study. All diets were rich in fruits, vegetables and lean meats consistent with food-based dietary recommendations. The three experimental diets (DASH, BOLD and BOLD+) contained similar amounts of total fat, SFA, monounsaturated fatty acids, polyunsaturated fatty acids and cholesterol. The HAD was higher in total fat, SFA, monounsaturated fatty acids, polyunsaturated fatty acids and cholesterol, and was lower in total fibre. The BOLD and DASH diets were matched for macronutrient composition. The BOLD+ diet was higher in protein (27% of total energy; 19% plant, 26% dairy, 42% lean beef and 12% other animal sources) as compared with the HAD (17%; 13% plant, 26% dairy, 12% lean beef and 49% other animal sources), DASH (18%; 20% plant, 31% dairy, 9% lean beef and 40% other animal sources) and BOLD (19%; 13% plant, 23% dairy, 53% lean beef and 11% other animal sources) diets, and lower in carbohydrate (45 vs 50–54%) (Table 1). A description of the food groups (and respective servings) fed has been published previously.^8^

**Table 1:** BOLD Study diets: energy (based on 2100 kcal meal plans) and nutrient composition (% of energy)^a^^b^^c^

*Nutrient targets, kcal % (g)*	*Diets*			
	*HAD*	*DASH*	*BOLD*	*BOLD+*
Calories	2097	2106	2100	2104
Protein (g)	17 (91.7)	18 (98.4)	19 (99.6)	27 (145.6)
Carbohydrate (g)	50 (268.1)	55 (298.3)	54 (287.4)	45 (243.7)
Fat (g)	33 (77.0)	27 (64.4)	28 (65.8)	28 (66.6)
Cholesterol (mg)	287	188	168	193
SFA (g)	12 (27.9)	6 (15.2)	6 (15.4)	6 (14.5)
PUFA (g)	7 (15.5)	8 (18.9)	7 (16.5)	7 (16.1)
MUFA (g)	11 (25.9)	9 (21.8)	11 (25.2)	12 (29.3)
Fibre (g)	24	36	32	38
*Micronutrients*				
Sodium (mg)	3243	2983	2712	3344
Potassium (mg)	3259	4247	3998	4417
Calcium (mg)	993	1140	936	1060
Magnesium (mg)	308	403	392	429
Lean beef, g/day	20	28	113	153

Abbreviations: BOLD, beef in an optimal lean diet; BOLD+, beef in an optimal lean diet plus additional protein; DASH, dietary approaches to stop hypertension diet; HAD, healthy American diet; MUFA, monounsaturated fatty acids; PUFA, polyunsaturated fatty acids; SFA, saturated fatty acids.

^a^Based on 2100 kcal per day.

^b^Average across a 6-day menu cycle.

^c^All values were determined using NUTRTIONST PRO (Axxya Systems LLC, Stafford, TX, USA).

Although matched for protein, the BOLD and DASH diets differed in the quantity of lean beef (Table 1). Select grade top round, chuck shoulder pot roast and 95% lean ground beef were used in the study. The meat was prepared via braising, grilling or frying (95% lean ground beef only), and was never cooked over an open flame in order to prevent charring.

A 6-day menu cycle was used throughout the study (1800–3600 kcal per day). All meals and snacks were prepared at the Metabolic Diet Study Center at Pennsylvania State University. Participants ate one meal per day (Monday–Friday) in the Metabolic Diet Study Center and their other meals were prepared and packed for off-site consumption. On weekends when the Study Center was closed, participants received a cooler that contained all of their meals and snacks for 2 days. Compliance with the prepared diets was monitored via self-report to document whether study foods/meals were omitted and/or replaced. Participants limited caffeinated beverages to 8 oz per day and alcoholic beverages to <2 servings per week. Participants were allowed to continue their current exercise regimen but were instructed not to increase or decrease duration or intensity during the study.

### Clinical assessments

Body weight was measured each weekday in the Metabolic Diet Study Center before eating that day’s meal and at each laboratory visit. Blood samples were collected after a 10–12-h fast. Serum and plasma aliquots were stored at −80 °C until time of analysis.

### Vascular health

Measures of vascular health were secondary end points in the BOLD Study. BP was assessed using a single measurement at the beginning of the study and at the end of each diet period before the baseline period of the endothelial function test (Dinamap Pro 100, Critikon, Milwaukee, WI, USA). Participants were seated with arm at heart level and appropriate cuff sizes were used. After a 12-h fast, EndoPAT2000 (Itamar Medical, Ltd, Caesarea, Israel) was used to measure relative changes in pulse wave amplitude before vs after occlusion.^10^ The EndoPAT technique is validated as a measure of endothelial function.^10, 11^ Two flexible probes were placed on the index fingers of the right (ischaemic) and left (control) hands, and a counter pressure was applied to both fingers continuously throughout the test. A BP cuff was placed on the right forearm and pulse amplitude was measured during baseline (5 min), occlusion (5 min) and reactive hyperaemia (5 min). Reactive hyperaemia index (RHI) was calculated as the ratio of the average pulse wave amplitude during hyperaemia (60 to 120 s of the post-occlusion period) to the average pulse wave amplitude during baseline in the occluded hand divided by the same values in the control hand and then multiplied by a baseline correction factor. We also calculated the Framingham RHI (FRHI) as described previously.^12, 13^

The EndoPAT device was used to generate the augmentation index (AI). The EndoPAT-generated AI measurement is determined from the baseline resting pulse wave. In stiff arteries, the pulse wave travels rapidly to the periphery where it encounters resistance at the peripheral arterioles, and the reflected wave augments central BP. Thus, higher AI indicates greater arterial stiffness. Proprietary software automatically identifies inflection points distinguishing the systolic peak and the reflected peak for the calculation of this ratio and converts it into a percentage (p1−p2/p1 × 100).^14^ EndoPAT-derived AI measures correlate well with AI measures from other devices.^15^ AI can be adjusted to a heart rate of 75 beats per min to correct for the independent effect of heart rate on AI measurement; both AI and AI at 75 beats per min are reported.

### Statistical analysis

All statistical analyses were performed using SAS (Version 9.2; Statistical Analyses System, Cary, NC, USA). Two sample *t*-tests were used to determine significant differences between genders at baseline for each outcome variable. The residuals for each variable were used to assess normality. Logarithmic transformations were used for non-normally distributed variables (AI and RHI). The mixed models procedure (PROC MIXED) was used to test the effects of diet and order on the outcome variables. A repeated analysis of covariance (repeated for diet) was used with age, weight and baseline values as covariates. Tukey–Kramer adjusted *P*-values were used to determine whether the differences between the diets for outcome variables were significant (*P*<0.05).

## Results

**Figure 1:**
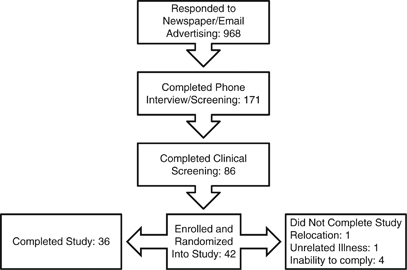
Recruitment flow diagram.

Figure 1 presents information about the number of subjects who responded to the study advertisements (*n*=968); completed the phone interview/screening (*n*=171); completed the clinical screening (*n*=86); and enrolled in the study (*n*=42). The vast majority of potential subjects were excluded based on the inclusion and exclusion criteria of the study after they completed the screening processes. Some individuals who met the study eligibility criteria elected to not participate because of the requirements imposed by the study. During the study, one subject dropped out because of a job change and relocation, one to an unrelated illness and four because of an inability to adhere to the dietary protocol (the latter occurred within the first week of the study). One participant was on BP-lowering medication for the duration of the study and was excluded in the analysis. There were 36 subjects included in the final analysis (Figure 1).

Baseline subject characteristics are presented in Table 2. Females had a significantly higher FRHI (0.79 vs 0.43; *P*=0.04) compared with males. Males had a significantly greater body mass index (27.3 vs 24.8 kg m^−2^; *P*=0.02) compared with females (Table 2). There were no gender differences in response to any of the dietary treatments. Subject adherence to the prescribed diets was 93% according to daily self-reporting forms. Body weight was maintained during the diet periods within ±2.2 kg. The metabolic status (lipids, glucose, insulin and C-reactive protein) of these subjects has been described previously.^8^

**Table 2:** Baseline characteristics of study participants (*n*=36)^a^

*Characteristic*	*Males (*n*=15)*	*Females (*n*=21)*
Age (years)	49±1.8 (39–63)	50±2.0 (45–97)
BMI (kg m^−2^)	27.3±0.7 (19.4–35.5)	24.8±0.5 (19.4–35.5)^b^
SBP (mm Hg)	124±2.6 (111–143)^b^	112.4±3.2 (94–150)
DBP (mm Hg)	72±2.0 (60–85)	66±2.6 (45–97)
RHI	1.98±0.18 (1.4–3.6)	2.33±0.13 (1.1–3.0)
FRHI	0.43±0.12 (0.2–1.4)	0.77±0.10 (0.01–1.4)^b^
AI	7.33±5.1 (−20.4–53.2)	18.0±5.08 (−17.6–49.4)

Abbreviations: AI, augmentation index; BMI, body mass index; BPM, beats per min; DBP, diastolic blood pressure; FRHI, Framingham reactive hyperaemia index; RHI, reactive hyperaemia index; SBP, systolic blood pressure.

^a^Mean ±s.e.m. (range). Baseline values were measured before consuming any study food.

^b^Two-sample *t*-test was used to determine significant (*P*<0.05) differences between genders (SAS version 9.2; SAS Institute Inc., Cary, NC, USA).

### Blood pressure

Systolic blood pressure (SBP) was significantly reduced following the BOLD+ diet compared with the HAD (*P*<0.01); no other significant reductions in SBP were observed. There were no significant changes in DBP on the DASH, BOLD or BOLD+ diets.

### Endothelial function and vascular stiffness

The RHI and FRHI scores did not differ among diets (Table 3). AI significantly decreased following the BOLD diet compared with the HAD, DASH and BOLD+ diets (Table 3).

**Table 3:** Effect of diet on blood pressure, endothelial function and vascular reactivity*

	*HAD*	*DASH*	*BOLD*	*BOLD* ^+^
Weight, kg	74.1±2.3	73.8±2.3	73.7±2.3	74.1±2.3
SBP, mm Hg	115.7±1.9^a^	112.9±1.9^a^	114.0±1.9^a^	111.4±1.9^b^
DBP, mm Hg	69.8±1.5	69.1±1.5	69.4±1.5	69.1±1.5
FRHI	0.65±0.05	0.66±0.05	0.64±0.05	0.62±0.05
RHI	2.21±0.09	2.19±0.10	2.31±0.09	2.13±0.11
Heart rate, BPM	58.01±0.74	58.30±0.74	60.03±0.74	59.30±0.75
AI**	14.47±3.6^a^	13.56±3.3^a^	10.37±3.0^b^	13.48±3.0^a^
AI @75 BPM	2.84±2.2	3.87±2.2	1.23±2.2	4.61±2.2
Older, AI**	21.97±5.0	22.71±2.4	19.62±4.1	17.72±4.1
Younger, AI**	6.08±4.5^a^	3.86±3.7^a^	0.03±26^b^	8.48±4.4^a^

Abbreviations: AI, augmentation index; BOLD, beef in an optimal lean diet; BOLD+, beef in an optimal lean diet plus additional protein; BPM, beats per min; DASH, dietary approaches to stop hypertension diet; DBP, diastolic blood pressure; FRHI, Framingham reactive hyperaemia index; HAD, healthy American diet; RHI, reactive hyperaemia index; SBP, systolic blood pressure.

The MIXED procedure (version 9.2; SAS Institute Inc., Cary, NC, USA) was used to test the effects of diet. Values in the same row with different superscripts (a, b) are significantly different, adjusted *P*<0.05.

*All values are mean±s.e.m.

**Raw values reported. Data were log transformed to achieve normality when testing for significant differences. Older females ⩾55 years and males ⩾45 years.

A significant interaction was observed between subject age and diet for AI. To further explore this interaction, subjects were grouped according to whether their age was a risk factor for CVD (females ⩾55 years and males ⩾45 years).^16^ This secondary analysis revealed that AI was significantly reduced on the BOLD diet in younger participants, but not older individuals.

## Discussion

The BOLD Study is the first controlled clinical trial to show that a moderate protein diet (based on the DASH eating plan) that emphasized lean beef (113 g per day) as the main protein source reduced SBP in normotensive individuals when compared with a healthy control diet that was lower in protein and higher in carbohydrate and saturated fat. The BOLD+ diet contained 10% more calories from protein and reduced SBP by 4.2 mm Hg versus HAD, whereas the BOLD and DASH diet elicited nonsignificant reductions of 1.6 and 2.8 mm Hg, respectively.

In the OmniHeart trial, the high-protein diet that had a comparable macronutrient profile to the BOLD+ diet resulted in a −9.5 mm Hg reduction in SBP from baseline.^17^ The difference in the magnitude of BP reductions in the present study and the protein diet evaluated in the OmniHeart trial could be due, in part, to the participants studied. Specifically, participants in the BOLD Study were normotensive (mean baseline SBP, 116±3.6 mm Hg), whereas participants in the OmniHeart trial were pre-hypertensive (mean baseline SBP, 131.3±10.8 mm Hg). In the BOLD+ diet, as in the higher protein OmniHeart diet, increases in total protein (from either animal or plant protein) suggest that the BP reductions reflect a total protein effect or the synchronous reduction in carbohydrates.

Compared with the original DASH trial (which lowered SBP by −3.5 mm Hg),^18^ the BOLD and DASH diets yielded similar, yet nonsignificant changes in SBP versus HAD (−1.9 and −2.8 mm Hg, respectively). The minor differences in response to the DASH diet and the similarly designed BOLD diet in our study may be because of the normotensive status of the study population compared with the pre-hypertensive/hypertensive participants in the DASH trial. Individuals with hypertension have greater reductions in BP following a heart healthy diet,^19^ as well as after weight loss^20^ compared with their normotensive counterparts. In addition, our study had far fewer subjects (*n*=36) than the DASH trial (*n*=459), and this could explain the lack of statistical significance for the SBP change observed. The differences in the response observed in the BOLD Study may also be due in part to differences in total and saturated fat between the control diet used in the initial DASH study (total fat=37%; SFA=16% of total energy) and the HAD (total fat =33%; SFA=12% of total energy).^21^

In addition to the aforementioned protein effect (or carbohydrate reduction), potassium, magnesium, sodium and calcium are minerals of importance with respect to their role in modulating BP.^22^ Sodium and calcium intakes were similar for the HAD and BOLD+ diets (Table 1). Potassium and magnesium levels were lower in the HAD compared with the BOLD+ diet. The BOLD+ diet provided similar amounts of potassium and magnesium compared with the original DASH study (4415 and 480 mg per day, respectively),^18^ and potassium levels were similar to those recommended by the American Society of Hypertension (4700 mg per day).^2^ A 2006 Cochrane review on magnesium supplementation for the treatment of high BP did not find evidence to support a causal relationship.^23^ A systematic review by Dickinson *et al.*^24^ found that magnesium supplementation (0.2–1.0 g per day) reduced SBP by 1.3 mm Hg, but this was nonsignificant. Therefore, we believe that it is unlikely that the 121 mg per day difference in magnesium between the HAD and BOLD+ diet significantly contributed to the reduction in SBP. We hasten to add, however, that in a diet that is also increased in other nutrients that are shown to lower BP, a small increase in magnesium may contribute to a BP-lowering effect.

It is also unlikely that dietary fibre is responsible for the reduction in SBP. A meta-analysis of 25 randomized controlled trials found no effect of dietary fibre intake on SBP in normotensive individuals.^25^ Thus, the 14 g per day increase in dietary fibre between the HAD and BOLD+ diet likely did not influence SBP in normotensive subjects in the BOLD Study. Although the separate effect of fibre and select minerals does not fully explain the BP-lowering effects, there is most likely an effect of the synergy of these changes, as seen in the original DASH trial. Taking this into account, we still propose that the primary explanation for the changes in SBP in our normotensive study population was most likely because of the increase in total protein (from a variety of protein sources, including lean beef) that also led to a slight decrease in carbohydrate. Further studies are needed to resolve this question.

On the BOLD diet, AI was significantly reduced compared with the HAD, despite no significant changes in DBP or SBP. In addition, AI was not significantly correlated with DBP (*R*=0.063, *P*=0.4) or SBP (*R*=0.15, *P*=0.06). However, there is conflicting evidence regarding the relationship between peripheral BP and AI.^26, 27, 28^ Hamburg *et al.*^13^ hypothesized that the discrepancy in the relationship between peripheral arterial tonometry measures, like AI, and systemic BP may be because of the limited effect of systemic BP on the distal microcirculation. This might be the case in the BOLD Study as significant improvements in AI were observed in the BOLD but not BOLD+ diets, whereas SBP was reduced in the BOLD+ but not BOLD or DASH diets.

AI and age are also related.^29^ Our results are consistent with this finding as a secondary analysis revealed a significant reduction in AI following the BOLD diet only in younger participants (males <45 years and females <55 years). This suggests that the arterial stiffness associated with ageing^30^ was not modified by the BOLD diet. Thus, dietary interventions designed to improve vascular reactivity may need to be initiated earlier in life to have a significant effect.

The mechanism accounting for the significant reduction in AI following the BOLD diet compared with the HAD, DAS and BOLD+ diets is unclear. This is one of the first controlled-feeding studies to measure the impact of different macronutrient composition (as well as protein sources) on AI. The improvements in AI observed for the BOLD diet underscore the need to better understand the effects of diet and protein (that is, quantity and source) on vascular elasticity.

The endothelial function results of the current study (as assessed by EndoPat) agree with those of a 35-day controlled-feeding intervention conducted by Vega-López *et al.*^31^ who found no effect of increasing the dietary lysine/arginine ratio (0.7 to 1.4; a common measure of the animal/plant protein ratio) of a low SFA diet (<7% total calories) on flow-mediated dilation or peripheral arterial tonometry (precursor to RHI measurement provided by EndoPat 2000). Al-Solaiman *et al.*^32^ and Hodson *et al.*^33^ also found no changes in endothelial function in healthy individuals following the DASH diet.

There were three potential limitations of the vascular health end points in the BOLD Study. One potential limitation was that BP was only measured once at baseline and at each end point visit. A minimum of two measurements taken 1 min apart is the preferred method to reduce measurement error. In addition, the null finding for RHI and FRHI may have been influenced by the menstrual phase that was not controlled for in the present study; however, we also did not observe any significant changes in RHI or FRHI in male subjects. Finally, in this study, macronutrient intakes and other nutrient guidelines were set based on percent of total calories or in the context of the base diet of 2100 calories. Although the calorie levels for individuals were adjusted up or down based on the energy needs required to maintain a participant’s weight, nutrients such as fibre, potassium, sodium and magnesium were also increased or decreased but not always in an exact proportional manner. Because it is not known whether meeting the exact nutrient targets established in the DASH study for different calorie levels is important, this could be one possible reason why a statistically significant BP reduction was not observed for the DASH and BOLD diets in the present study.

The BOLD diet was the only diet that significantly reduced AI, and the BOLD+ diet was the only diet that significantly reduced SBP. These findings suggest that heart-healthy diets containing different amounts of macronutrients including those contained in lean beef (that is, primarily protein) can positively affect vascular health, although via potentially different mechanisms. It has been suggested previously that increased dietary plant protein (versus animal protein) may be responsible for the protein-associated reductions in BP.^34^ However, the present findings show that a variety of protein sources including lean beef can also be used to increase total dietary protein in a heart-healthy diet as a strategy to reduce SBP in normotensive individuals. Thus, increasing total dietary protein (or decreasing dietary carbohydrate) in combination with a diet rich in fruits, vegetables, fibre and low-fat dairy appears to play an important role in reducing SBP.

We had previously shown^8^ that DASH, BOLD and BOLD+ each lowered cholesterol similarly compared with HAD and that these diets had no effect on fasting glucose and insulin levels. Thus, it is unlikely that the differential effects of DASH, BOLD and BOLD+ on vascular status are mediated by changes in lipids, glucose or insulin.

Further controlled clinical trials are needed to elucidate the role and mechanism(s) of action of both protein sources and quantity on BP and vascular health in normotensive and hypertensive individuals.